# Diagnosing Celiac Disease: Towards Wide-Scale Screening and Serology-Based Criteria?

**DOI:** 10.1155/2019/2916024

**Published:** 2019-08-06

**Authors:** Alina Popp, Laura Kivelä, Valma Fuchs, Kalle Kurppa

**Affiliations:** ^1^Center for Child Health Research, Tampere University and Department of Paediatrics, Tampere University Hospital, Tampere, Finland; ^2^Institute for Mother and Child Health Bucharest, University of Medicine and Pharmacy “Carol Davila”, Bucharest, Romania; ^3^University of Helsinki and Helsinki University Hospital, Children's Hospital and Pediatric Research Center, Helsinki, Finland; ^4^Celiac Disease Research Center, Tampere University, Tampere, Finland; ^5^The University Consortium of Seinäjoki, Seinäjoki, Finland

## Abstract

Celiac disease is one of the most common food-related chronic disorders in children. Unfortunately, this multifaceted disease is challenging to recognize and remains markedly underdiagnosed. Screening of either known at-risk groups or even the whole population could increase the suboptimal diagnostic yield substantially. Many recent guidelines recommend screening of at least selected risk groups, but more wide-scale screening remains controversial. The increasing prevalence of celiac disease and the development of autoantibody assays have also led to a gradual shift in the diagnostics towards less invasive serology-based criteria in a subgroup of symptomatic children. The main open questions concern whether these criteria are applicable to all countries and clinical settings, as well as to adult patients. On the other hand, widening screening and the mistaken practice of initiating a gluten-free diet before the appropriate exclusion of celiac disease increase the number of borderline seropositive cases, which may also challenge the classical histopathological diagnostics. Sophisticated diagnostic methods and a deeper understanding of the natural history of early developing celiac disease may prove useful in these circumstances.

## 1. Introduction

With a prevalence of up to 1–3%, celiac disease is one of the most common chronic gastrointestinal diseases [1–3]. It is evident that the diagnostics of such a frequent condition should be effective and practical. Unfortunately, the heterogeneous clinical presentation makes the disease difficult to recognize, and currently the great majority of affected individuals remain undiagnosed, leaving them vulnerable to long-term complications [3, 4]. The most effective means of improving the diagnostic yield would be to screen known at-risk groups or even the whole population. The development of advanced serological tests has made screening rather straightforward, but the overall benefits of this approach remain a matter of debate [5]. Particularly controversial issues are the treatment of asymptomatic screen-detected individuals, the optimal age for rescreening, the optimal rescreening frequency, and the utilization of genetic testing to further delineate the susceptible cohort.

Traditionally, the diagnosis of celiac disease has been based on the demonstration of mucosal injury in duodenal biopsy. This invasive approach has been considered necessary to ensure the diagnosis before starting a demanding gluten-free diet. However, the high specificity of modern serological tests and the desire to reduce the need for invasive investigations led to the release of new criteria by the European Society for Paediatric Gastroenterology, Hepatology, and Nutrition (ESPGHAN) in 2012, which allow for the first time a noninvasive approach to diagnosis in a subgroup of children [6]. Although a huge leap forward, these guidelines paradoxically created new challenges, as they are currently not accepted in all countries and were not drawn up for adults [7, 8]. Furthermore, even if the novel approach was adopted more widely, biopsy would still be needed in individuals with low positive serology, which are often diagnostically the most problematic cases. In fact, the number of such individuals is likely increasing due to more active screening.

In this review, we provide an overview of the current concepts of the diagnostics of celiac disease in children and adults. The main topics discussed are the possibilities for improving the suboptimal diagnostic yield and efforts to provide more unified diagnostic guidelines in the light of the most recent scientific evidence. Furthermore, we discuss the future directions in diagnostics, particularly concerning early developing celiac disease with minor or no histopathological changes and otherwise challenging cases.

## 2. Diagnostic Approach: From Case Finding towards Screening

The phenotype of celiac disease extends from varying gastrointestinal and extraintestinal complaints to an apparent lack of symptoms [9]. This variation makes recognition of the disease challenging, and currently the majority of affected children and adults remain undiagnosed (Table 1). The main approaches to detect untreated celiac disease are active case finding based on clinical symptoms and signs and targeted screening of at-risk groups, such as the relatives of celiac disease patients and subjects with certain other autoimmune diseases. However, there are major differences in the diagnostic approach between and even within countries, and this is also reflected in the inconsistencies between the true population-based prevalence of celiac disease and the number of actually diagnosed patients (Table 1).

### 2.1. Case Finding

Case finding is, in theory, an effective approach to find at least those patients with a characteristic clinical presentation. However, only those who seek medical help because of their symptoms or other clinical signs can be found, which requires activity from the patients themselves. Furthermore, medical practitioners should be alert to the possibility of celiac disease behind the various complaints they encounter in daily practice. Unfortunately, this seems to be very challenging in the case of celiac disease. It has been observed that up to 85% of patients eventually found by screening have suffered from unrecognized symptoms for some time—even for several years—before the diagnosis [10–14]. The situation is further complicated by the low predictive value of even “typical” gastrointestinal symptoms for celiac disease [15, 16].

### 2.2. Screening: Current Approaches and Open Questions

There is a clear need for more effective diagnostic approaches rather than relying on ineffective case finding. The development of practical serological tests in recent decades has enabled easier noninvasive screening, but the matter of who should be screened is all but clear [5, 17]. Celiac disease fulfills most of the World Health Organization's general criteria for screening, but further studies are needed, particularly regarding the cost-effectiveness of screening and the natural history of clinically unrecognized patients [17]. The main issue is whether the benefits of an early diagnosis overcome the costs, laboriousness, and social burden of a gluten-free diet [5].

One argument against screening is the low risk for complications in unrecognized celiac disease patients. However, as already mentioned, many screening-detected subjects actually suffer from unrecognized symptoms. Moreover, even truly asymptomatic patients might be at risk for ill-health and long-term complications if left untreated [14, 18–22]. Particularly in children, many complications—such as dental enamel defects, poor height gain, and reduced bone accrual—may remain permanent if not detected early enough [23–25]. Furthermore, if the disease remains untreated until adulthood, there is an increased risk, e.g., for infertility, refractory celiac disease, and even small-bowel lymphoma [26, 27]. Delayed diagnosis may also predispose to reduced quality of life, the incremental use of medicines, and persistent symptoms even on a gluten-free diet [28, 29].

A critical aspect regarding the rationale of screening is the patient's willingness to adhere to a life-long treatment. There has been concern that asymptomatic patients in particular would have poor motivation to maintain the diet with no apparent clinical benefits. Reported dietary adherence in screen-detected patients has varied from 23% to 93% [12, 14, 30–36]. In more recent studies, the general tendency has been better adherence, possibly reflecting the increasing knowledge and better availability of gluten-free products in grocery stores and restaurants [31, 37–41]. In fact, nowadays even asymptomatic screen-detected patients may show excellent dietary adherence [14, 42].

The most recent guidelines recommend screening for celiac disease in selected at-risk individuals (Table 2), although the actual implementation of the screening in clinical practice varies [43]. However, even with targeted screening, a substantial percentage of affected individuals will remain unrecognized [44]. The only option to find almost all celiac disease patients would be to screen the entire population or, alternatively, subjects with a genetic predisposition. This approach would still leave the question of the frequency of repeated screening after negative results, as celiac disease may develop at any age [2, 45]. Furthermore, wide-scale screening would likely reveal a significant number of seropositive subjects who do not fulfill the current diagnostic criteria. At present, the prognosis and benefits of the early diagnosis and treatment of such cases are poorly known [46–51].

## 3. Changing Guidelines and Development of Diagnostic Tools

### 3.1. Histology and Serological Tests

During the past 70 years, the diagnostics of celiac disease has evolved from symptom-based deduction to the use of sophisticated serological and histological methods. The development of biopsy techniques, followed by the description of duodenal injury, have been critical milestones [52, 53]. Before the 1970s, histology was the only diagnostic method in all age groups [54]. A less invasive approach for case finding could be attained by using serum antibodies, the first of which were antigliadin antibodies with moderate sensitivity and specificity [55, 56]. More specific autoantibodies to reticulin and—particularly from the 1980s—endomysial antibodies (EmA) proved to be valuable tools for initial screening [57–59]. The identification of transglutaminase 2 as the autoantigen recognized by EmA [60] enabled practical ELISA tests for the detection of transglutaminase 2 antibodies (TG2ab) [61].

There have also been improvements in the histopathological assessment. The original biopsy capsule was gradually replaced by endoscopic duodenal sampling. In 1992, Marsh introduced the now widely used grouped classification for histological injury [62], and a modified version of this grading was later advocated by Oberhuber [63]. For these classifications, the histological injury is divided for practical purposes into three classes: infiltrative (Marsh 1), hyperplastic (Marsh 2), and atrophic (Marsh 3) lesions. In the Oberhuber classification, stage 3 is further divided into subclasses 3a, 3b, and 3c. The more quantitative assessment of the mucosal damage using villous-height crypt depth measurement was introduced in the early 1980s and later further improved [64, 65]. At present, however, this methodology is used mostly in research settings.

### 3.2. Evolving Guidelines towards a Less Invasive Diagnostic Approach

With some modifications, the ESPGHAN 1990 criteria for celiac disease remained the basis of practically all pediatric and adult diagnostic guidelines until 2012 [6–8, 66–70]. Demonstration of the characteristic histological lesion, followed by the resolution of symptoms on a gluten-free diet, allowed the establishment of the diagnosis, with positive serology giving further support to the diagnosis [67]. In the early 2000s, the testing of TG2ab came to the forefront in initial case screenings in both children and adults, although histological confirmation was still required [7, 71]. Groups at risk for celiac disease were also increasingly recognized, and their low-threshold screening was recommended.

In 2012, the new EPSGHAN diagnostic criteria were launched [6]. The main driving forces for the revision were the necessity for general anesthesia for invasive endoscopy in children and the excellent positive predictive value of modern serological tests, particularly high tTGab values (Table 3) and positive EmA. For the first time, the revolutionary guidelines allowed diagnosis without biopsy in specific circumstances; i.e., for symptomatic children with tTGab values > 10× the upper limit of normal (ULN), positive EmA, and the presence of the at-risk human leucocyte antigen (HLA) DQ2/DQ8 haplotype [6]. Recent prospective studies have provided strong support for the accuracy of these criteria [72, 73]. There is growing evidence that the nonbiopsy diagnostic approach could be applied reliably also for asymptomatic children [74–76] and without mandatory genetic testing [72]. In adults, histological evaluation has remained the cornerstone of the diagnosis, excluding the recently published Finnish guidelines that allow a nonbiopsy approach in some patients, regardless of their age [68].

## 4. Challenges with the Diagnostic Criteria and Future Directions

### 4.1. Technical Challenges

A major challenge in the current diagnostics is the lack of standardization in tTGab kits. This is particularly problematic for the nonbiopsy criteria, as the resulting incomparability between the assays may even predispose to misinterpretations [77, 78]. In order to err on the side of safety, ESPGHAN recommends using only tTGab tests with an appropriate calibration curve [6]. Furthermore, the rather high ULN cutoff value and the requirement of HLA and EmA testing were included partly to control the assay variation. As mentioned, it might be possible to omit HLA testing in the future [72], and the role of EmA could also be questioned. Although EmA is highly specific, the required immunofluorescence method is laborious and not universally available. By applying well-validated tTGab assays, it might be possible to abandon EmA and also lower the diagnostic ULN threshold [72, 79].

Histopathology might not be as good a diagnostic reference standard as previously thought. The mucosal lesion can be patchy, the quality of the biopsies is often inadequate, and duodenal injury is not fully specific to celiac disease [80, 81]. In order to improve the diagnostic yield, the current recommendation is to take at least four biopsies from the distal duodenum and one from the bulb [6, 66, 82, 83]. However, due to the lower specificity, the added value of the duodenal bulb biopsy is controversial, and caution is needed when a diagnosis of celiac disease is based solely on bulb samples [84]. In addition, even if representative biopsies are obtained, their correct handling and orientation are often challenging and prone to mistakes [65]. Accordingly, several studies have shown poor intra- and interobserver agreement between pathologists when applying a grouped histological classification [85–87].

### 4.2. Lack of Unified Guidelines

One of the main challenges with the current diagnostic criteria is their age- and country-related variation [6, 8, 69]. Despite the aforementioned problems, duodenal histopathology as the gold standard used to be the unifying feature of all the guidelines [67]. This changed radically when the ESPGHAN criteria introduced the possibility of omitting endoscopy for some European children [6], while the biopsy remains mandatory, e.g., in the USA [7]. These discrepancies might be explained to some extent by the different health care systems [88]. In addition, most of the studies on this issue have been made in Europe and, for unclear reasons, studies from North America have reported the inferior accuracy of tTGab tests (Table 3). As it is unlikely that children differ significantly between the continents, and since joint guidelines exist for many other diseases [89, 90], unified criteria for celiac disease would seem reasonable.

Another issue is the acceptance of serology-based diagnoses of celiac disease in adults by physicians. Only one of the current guidelines makes a clear statement on this issue; it does not support the taking of routine duodenal biopsies to reconfirm the diagnosis in adults when the diagnosis has been set strictly according to the ESPGHAN criteria [91]. This issue is particularly important in the transitional period from childhood to adulthood, when some young patients and/or their physicians may question the initial diagnosis [92]. To avoid confusion and the unnecessary repetition of diagnostic procedures, general acceptance—or preferably unified adult and pediatric criteria—is important.

Recent studies have given evidence that the nonbiopsy criteria would apply also to adults [79, 93, 94], but many experts remain cautious [95, 96]. One fear is the misuse of the criteria by general practitioners [96–98]. However, there is evidence that accurate diagnostics can be achieved by education and close collaboration with primary care [94, 99]. Another feared consequence of omitting endoscopies is missing a coexisting disease or complication, such as refractory celiac disease or malignancy [93, 96]. In practice, however, this does not seem to be a major problem, although more evidence is called for [79, 93, 100, 101]. In general, the new guidelines do not aim to ban biopsies, but rather to offer the option for diagnosis without endoscopy in definite cases [6]. Endoscopy would still be preferable if red flag symptoms such as bloody stools, dysphagia, or severe weight loss appear, or if there is incomplete clinical recovery [6, 79, 101].

### 4.3. Challenging Diagnostic Scenarios

Despite the tendency towards less invasive approaches, duodenal biopsy will likely remain a part of celiac disease diagnostics for quite some time. The main problem with serology is that the specificity decreases with lower antibody values [72, 79]. Unfortunately, such patients are usually also histologically the most problematic cases, as they may present only with mild or patchy duodenal changes [46, 80]. In these circumstances, it is important to confirm that all stages of duodenal sampling and histological analysis have been done correctly [65, 102]. The more quantitative measurement of architectural changes, e.g., by applying validated duodenal histomorphometry, might also prove useful [65].

The widening use of screening can be expected to increase the number of patients detected with early stage celiac disease and morphologically normal villi [46, 49, 103]. There is evidence that seropositive individuals may suffer from symptoms and signs already at this point and benefit from a gluten-free diet [46, 49, 50, 104], indicating that the whole definition of celiac disease might require reevaluation. Nevertheless, many such individuals are asymptomatic and do not develop duodenal lesions even during a long-term follow-up [49, 105–107]. It is essential to learn more about the natural history of early developing celiac disease in order to discern cases that would truly benefit from early diagnosis [108].

Another challenge in the differential diagnosis of patients with borderline or negative serology is brought by the now common practice of initiating a gluten-free diet before appropriate diagnostic investigations [109, 110]. It still might be possible to establish the diagnosis using sophisticated techniques, e.g., determination of small-bowel mucosal *γδ*+intraepithelial lymphocytes and celiac disease-specific tTG-targeted IgA deposits [46, 111–114]. Genetic testing and recently introduced innovative methods, such as HLA-DQ-gluten tetramer-based assays, might further help to exclude or confirm the presence of celiac disease [115].

### 4.4. Prevention of Celiac Disease?

In the future, it might even be possible to proceed a step further, as several ongoing prospective birth cohort studies are steadily providing a deeper understanding of the early development of celiac disease [116–120]. Increasing information about the disturbed balance of genetics and environmental factors in celiac disease might offer possibilities for the early detection of high-risk children, and perhaps even provide means for primary prevention [116–119, 121].

## 5. Conclusions

Owing to the high prevalence of celiac disease, even minor changes in the diagnostic approach may have substantial effects on health care and society. It is evident that the only effective way to improve the currently unsatisfactory diagnostic yield is more widespread screening. Such an approach could be expected to prevent ill-health and severe complications in the long run, but it must be backed up with high-quality scientific evidence. Effective implementation of intensified case finding and screening also requires close collaboration with primary care and general practitioners, who are responsible for the first-line diagnostics.

Simultaneously with the increasing prevalence, the diagnostic criteria of celiac disease are currently undergoing revolutionary changes. At present, the serology-based diagnosis is limited to a minority of patients—i.e., mainly to symptomatic European children. This may cause problems, e.g., in the acceptance of the diagnosis in different countries and after the transition from pediatric to adult care. Since there is no apparent biological reason for the age- and site-related differences in the criteria, it would be desirable for more unified evidence-based global guidelines for celiac disease to be formed.

Notwithstanding the increasing tendency towards noninvasive diagnostics, biopsy will likely play an important role also in the future, particularly in individuals with low and/or borderline positive serology. In fact, the number of these cases will likely increase significantly concurrently with the widening screening and earlier testing. Novel sophisticated diagnostic tools may offer better possibilities for differential diagnosis in these often challenging situations. Open questions and issues remain concerning the natural history of these often asymptomatic individuals, particularly whether they should be diagnosed and treated with a gluten-free diet.

## Figures and Tables

**Table 1:** True prevalence of celiac disease based on screening studies and the proportion of clinically unrecognized patients.

Reference and year	Country	Diagnostic criteria	Prevalence (%)	Unrecognized (%)
*Children*				
Mäki et al., 2003 [122]	Finland	Biopsy	1.1	75.9
Tommasini et al., 2004 [123]	Italy	Biopsy	1.1	94.5
Myléus et al., 2009 [2]	Sweden	Biopsy	2.9	69.3
Mustalahti et al., 2010 [124]	UK	Seropositivity^a^ or biopsy	0.9	94.4
Laass et al., 2015 [125]	Germany	Seropositivity^a^	0.8	91.7
*Adults*				
West et al., 2003 [126]	UK	Seropositivity^a^	1.2	95.7
Lohi et al., 2007 [1]	Finland	Seropositivity^a^	2.0	74.9
Mustalahti et al., 2010 [124]	Germany	Seropositivity^a^ or biopsy	0.3	93.3
Mustalahti et al., 2010 [124]	Italy	Seropositivity^a^ or biopsy	0.7	97.1
Rubio-Tapia et al., 2012 [127]	USA	Seropositivity^a^	0.7	90.1
Fukunaga et al., 2018 [128]	Japan	Biopsy	0.1	100

^a^Positive tissue transglutaminase and/or endomysial antibodies.

**Table 2:** Recommendations on screening for celiac disease according to the most recent diagnostic guidelines.

Reference	Organization	Age group	Screening recommendation
Downey et al., 2015 [66]	NICE	Children and adults	T1D, autoimmune thyroidal disease, and family risk
Ludvigsson et al., 2014 [8]	BSG	Adults	T1D, irritable bowel syndrome, Down syndrome, and family risk
Rubio-Tapia et al., 2013 [69]	ACG	Children and adults	Symptomatic T1D and family risk
Husby et al., 2012 [6]	ESPGHAN	Children	T1D, autoimmune thyroidal and liver diseases, IgA deficiency, family risk, and Down, Turner, and Williams syndromes
Hill et al., 2005 [7]	NASPGHAN	Children	T1D, autoimmune thyroidal and liver diseases, family risk, and Down, Turner, and Williams syndromes

ACG: American College of Gastroenterology; BSG: British Society of Gastroenterology; ESPGHAN: European Society for Paediatric Gastroenterology, Hepatology, and Nutrition; NASPGHAN: North American Society for Pediatric Gastroenterology, Hepatology, and Nutrition; NICE: The National Institute for Health and Care Excellence; T1D: type 1 diabetes.

**Table 3:** Studies assessing the positive predictive value (PPV) of high tissue transglutaminase antibody (tTGab) values in the diagnosis of celiac disease.

Reference	Cohort	Country	tTGab threshold	Number of tested assays	PPV (%)
*Children*					
Paul et al., 2018 [76]	157	UK	10x ULN	1^b^	100
Werkstetter et al., 2017 [72]	707	Multicenter	10x ULN	8	99.6–100^c^
Wolf et al., 2017 [73]	898	Germany	10x ULN	1	98.8
Smarrazzo et al., 2017 [129]	1,974	Multicenter	10x ULN	8	96.1
Elitsur et al., 2017 [88]	240	USA	10x ULN	1^b^	87.7
Trovato et al., 2015 [75]	286	Italy	10x ULN	1	91.0–92.5
Gidrewicz et al., 2015 [130]	17,505	Canada	10x ULN	1	92.8
*Adults*					
Efthymakis et al., 2017 [93]	234	Italy	10x ULN	2	97.6
Ganji et al., 2016 [131]	299^a^	Iran	10x ULN	1	100
Tortora et al., 2014 [101]	310	Italy	8.9x ULN	1	100

^a^Adults and adolescents. ^b^tTGab assay was not specified. ^c^Lowest obtained specificity when testing different diagnostic scenarios and excluding inconclusive patients. ULN: upper limit of normal; ND: no data.

## Abbreviations

EmA: Endomysial antibodies
ESPGHAN: European Society for Paediatric Gastroenterology, Hepatology, and Nutrition
HLA: Human leucocyte antigen
TG2ab: Transglutaminase 2 antibodies
ULN: Upper limit of normal.
